# The Role of Law Enforcement Officers/Police in Drug Prevention within Educational Settings—Study Protocol for the Development of a Guiding Document Based on Experts’ Opinions

**DOI:** 10.3390/ijerph18052613

**Published:** 2021-03-05

**Authors:** Ziad El-Khatib, Celina Herrera, Giovanna Campello, Elizabeth Mattfeld, Wadih Maalouf

**Affiliations:** 1 Drug Prevention and Health Branch, Prevention Treatment and Rehabilitation Section, United Nations Office on Drugs and Crime, P.O. Box 500, A-1400 Vienna, Austria; ziad.khatib@gmail.com (Z.E.-K.); giovanna.campello@un.org (G.C.); elizabeth.mattfeld@un.org (E.M.); 2 Department of Psychology, University of Hawai’i, Mānoa, HI 96822, USA; cherre24@hawaii.edu

**Keywords:** law enforcement officers, drug prevention, schools, educational settings, crime prevention

## Abstract

The United Nations Office on Drugs and Crime—World Health Organization International Standards on Drug Use Prevention—reflects the value of safe, nurturing and supportive social institutions around the lives of youths to benefit from the prevention of risky behavior extending beyond individually-developed resilience for healthy adolescent development. Schools are valuable social institutions to this effect and school safety and adolescent health outcomes can be threatened by drug use and violence. As such, collaborative, multi-level, evidence-based, developmentally sensitive, substance use prevention programs are imperative. The International Standards, in their latest version, did not reflect specific evidence of law enforcement officer-based programs with effect on drug use prevention, including in school settings. Nevertheless, the collaboration between law enforcement agencies and school-based substance use prevention programs continue to be the focus of research and policy. In this project, we aim to explore in more detail the role of law enforcement in preventing substance use in schools. We use mixed methods, including three phases: (i) scoping review on the best practices for effective law enforcement in school-based drug and crime prevention; (ii) interviews with experts, using the Delphi method, in substance use prevention and training law enforcement in school-based drug prevention; and iii) developing guidelines for law enforcement based on the findings. Initially, we identified a total of 17 papers that were categorized in four categories based on their results (negative or null effect *n* = 11 studies, positive effect *n* = 1 study, mixed effects *n* = 4 studies and indefinite conclusion *n* = 1 study). However, the authors of the studies with negative or null effect did recommend being cautious about these results due to the respective studies’ methodological limitations. The actual and perceived roles of police are largely unclear and/or variable. Therefore, clear outlines regarding law enforcement’s role within schools are crucial as one study showed that an officer’s role influences how they respond to student conduct. A secondary emergent theme from this review indicates that there is potential for positively impacting a youth’s perceptions of police through collaborative and engaging school-based programs. Currently the project is gradually moving to Phase II, where we are identifying the key experts based on scientifically published peer reviewed and grey literature/guidelines to investigate elements that make the role of law enforcement officers in school-based prevention more effective. Given the frequency with which policy makers around the world request information about the role of law enforcement in effective prevention efforts, guidelines on their roles within schools is a gap that needs to be filled. Such efforts would improve drug prevention in schools and better orient law enforcement’s role in drug prevention within educational settings.

## 1. Introduction

Schools represent valuable social institutions that play an essential role in the safe and healthy development of youths, including the prevention of substance use (e.g., tobacco, alcohol and marijuana) [1]. On the other hand, exposure and use of substances such as smoking, drinking and illicit drugs, continues to threaten healthy school environments as well as the safe development of youth. Therefore, the development of a holistic evidence-based substance use prevention strategy is a crucial step towards the support of children in schools. This strategy needs to be based on scientifically oriented programs embedded in a safe school ethos and environment. Law Enforcement Officers’ (L.E.O.) roles in such strategies, while frequently used, are not guided by a clear set of scientific data supporting the effective role they might potentially play to ensure such a safe school environment, which in turn, prevent substance use as well as other negative social and health consequences in schools.

### 1.1. Conceptual Framework

Substance use prevention, as well as safe schools’ environment, is influenced by several factors:

(i) individual factors: including age, education, income level, health, psychosocial problems. For example: students showing poor self-regulation, impaired control and impulsiveness are more likely to binge drink alcohol.
(ii) friends and family relationships: which includes the closest social circle, such as family members, peers, teachers and other close relationships. For example: students who are associated with deviant peers are more likely to use marijuana.
(iii) community: which is the settings in which social relationships occur, including schools, workplaces and neighborhoods. For example: living in neighborhoods with a long-term high rate crime is associated with higher risk for substance abuse.
(iv) society and broad social factors: including health, economic, educational and social policies that contribute to economic and/or social inequalities and cultural norms [2].

**Figure 1:**
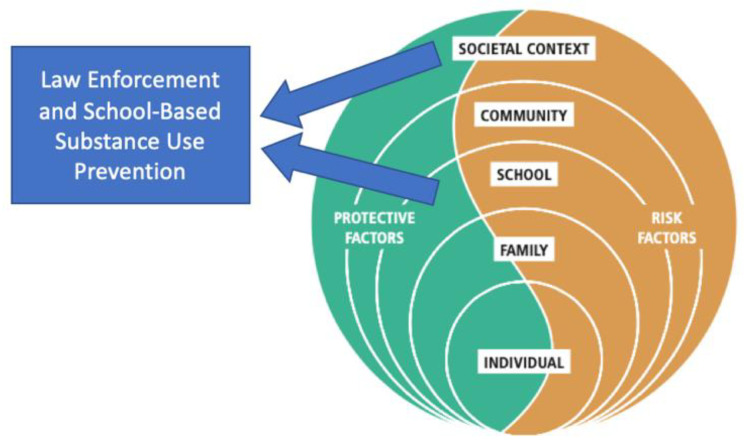
Categories of factors influencing the likelihood of a young person to engage in substance use.

Moreover, poor school safety is a threat element that increases the potential of substance use particularly amongst the most vulnerable of youths. The safety of the school environment is influenced by several factors that dynamically interact across the layers of the ecological system [3]. Therefore, substance use prevention in and around schools should include these different layers of a community’s ecological system to support substance prevention on a holistic scale. However, the incorporation of law enforcement officers in school-based substance use prevention is an ecological layer of the community. This has not been widely researched, despite its frequent report of use globally. Perceiving the role of law enforcement, for substance use prevention, has the potential to widen the spectrum of potential effect; this can carry within many different layers of the community ecology which in turn might support the prevention of substance use, including other social and health consequences, in schools (see Figure 1).

### 1.2. Adapted from the UNODC/WHO/UNESCO

Within such an approach, law enforcement components could integrate across the following areas: (1) Macro-Exo System: Implementation and enforcement of laws and policies on drug prevention; (2) Meso System: Promoting a healthy school environment that protects adolescents against substance use; (3) Micro System: Promoting positive attitudes toward law enforcement.

That being said, the role and the impact of law enforcement as an integral component of a school-based substance use prevention, including guidelines on the best practice, is still a research gap that remains to be filled. Effective guidelines can be developed when they are tailed in a larger health-centered system of drug use prevention. To this effect, the United Nations Office on Drugs and Crime (UNODC) has developed two guiding documents to support the strategic prevention response in the educational sector: (1) The UNODC/World Health Organization (WHO) International Standards on Drug Use Prevention and (2) UNODC/United Nations Educational, Scientific and Cultural Organisation (UNESCO)/WHO good policy and practice for education sector responses to the use of alcohol, tobacco and drugs [4,5]. However, within these guiding documents the specific role of L.E.O. was not clearly outlined, most likely given the existence of the aforementioned research gap.

The current efforts are to develop a guiding document on the role of L.E.O. in drug prevention in educational settings. There is a general indication, through the aforementioned ecological lens, that suggests law enforcement can play an important role in the field of school-based prevention. However, the extent of their role, their capacity to contribute and specific guiding documents reflecting on in what form might that be, when and how, are necessary for making the presence of law enforcement more effective.

Therefore, a literature review must be conducted to understand the ingredients of successes and opportunities (or failures) that have arisen from already existing school-based law enforcement programmes. This would help orient the efforts of law enforcement agencies to be more effective in the educational sector and also help better integrate their efforts within the larger pool of key stakeholders availing prevention responses nationally.

### 1.3. Context and Potentials for Law Enforcement in Schools

Teachers are engaged in and drive the development of a healthy school environment during the different grade levels and age groups of the students. During early school years, the main interventions involve classroom management or personal and social skills education programs. In early adolescence, the programmes include prevention education based on social competence and social influence, programs improved the mediating factor in the success or failure of students in the educational arena (known by the name of school attachment) [6], and indicated programs of early interventions to prevent escalation of the problem as well as environmental policies and interventions (namely school policies on substance use and alcohol and tobacco policies at the national or community level).

The law enforcement officers may be asked to contribute to or play a role in such modalities; however, there are no specific guiding documents to determine their capacity or guide their role. Therefore, we aim to bridge this gap by conducting a literature review informed by a group of experts.

The UNODC, WHO and UNESCO guidelines state prevention of substance use can benefit from an inter-disciplinary support. The strong involvement of the health and education ministries is key but also welcomes the close collaborations among other relevant ministries including the law enforcement agencies. Within the education sector, there are a range of actors and systems that constitute a rich education sector response [5]. Moreover, the Universal Prevention Curriculum 5 (UPC 5) reflects objectives of policies that support the involvement of law enforcement in response to substance use-related issues [7]. Furthermore, the UPC 5 highlights that involving law enforcement at the start of school-based prevention interventions can also aid in the collaborative drafting of inclusive and comprehensive school substance policies.

### 1.4. Current Role of Law Enforcement in Schools

The deployment of law enforcement officers, for drug use prevention, in schools is a relatively young practice (approximately since the 1980s). Usually, it is done in the form of recruiting police officers to schools on a full-time basis. Such police officers are known by the name of school resource officers (SRO) or education resource officers [8]. The aim of these police officers is to serve in various roles; this includes:

(i) Safety experts and law enforcers: for example, handling calls from schools and coordinating the response of other police resources, addressing crime and disorder problems and drug activities occurring in or around the school; making arrests and issuing citations on campus; contributing to investigations; taking action against unauthorized persons on school property; responding to off-campus criminal acts that involves students; being a liaison between the school and the police and providing information to students and school personnel about law enforcement matters;
(ii) Problem solvers and liaison to community resources: for example, crime prevention; taking initiative for community justice; being instrumental in changing the environment that can reduce crime in or around schools; supporting school policy development that address crime and their implementation process;
(iii) As educators, for example, teaching about policing as a career, criminal investigation, alcohol and drug awareness, gang and stranger awareness and resistance, crime prevention, conflict resolution, restorative justice, babysitting safety, bicycling, pedestrian and motor vehicle safety). Therefore, these assignments are becoming increasingly popular and the SRO programs are being encouraged, through governmental support.

There is a general indication in the literature that suggests law enforcement can potentially play an important role in the field of school-based prevention [8]. However, such an effective role has not been scientifically substantiated to be reflected in the UNODC/WHO International Standards on Drug Use Prevention [9]. Moreover, according to our knowledge there is a gap in guiding the effectiveness of the role of SROs to strategically prevent substance use, crime or violence which is important for the health promotion of the youth. Therefore, an extensive literature review must be conducted to understand the ingredients of successes and opportunities (or failures) that have arisen from already existing school-based law enforcement programs. This would help orient the efforts of law enforcement agencies to be more effective in the educational sector and also help better integrate their efforts within the larger pool of key stakeholders availing prevention responses nationally. A guiding document based on the existing literature and experiences globally, as well as networking and bridging of such experiences would be key in reaching this objective. This proposal ultimately aims at availing such a guiding document.

## 2. Materials and Methods

First, we have conducted an exploratory research study for the literature. The exploratory research is usually used when there is a limited evidence about the research area in question. Furthermore, the exploratory research findings are generally used to further research hypotheses [10]. Second, we planned the guidelines development through the following steps: (1) designing the concept, (2) targeting our key stakeholders, and (3) implementation and guideline development.

### 2.1. Scoping Review

Our search strategy was mixed between using keywords from both published literature and experts’ consultation (the keywords included: police and school, drug abuse intervention, crime prevention, school-based police officers and substance abuse). On the 11 October 2020, we had identified a total of 1230 articles; 408/1230 (33%) duplicates were removed from the initial articles leaving 822 articles. Further screening of the remaining articles showed that 152/822 of the articles did not meet the predetermined language criteria (not written in English language). Subsequent processing resulted in the removal of 610 articles that did not match the search criteria, and 18 papers had no full texts available. At the end of the screening process, only 12 relevant works of literature were included in this study. Additionally, we identified five additional articles, which makes the total number of articles 17.

Second we planned the guidelines development through the following steps [11]:

(a) Designing the concept: setting the procedural modality that will govern the development of the guiding document.
(b) Targeting our key stakeholders: this will include identification and direct contact of the key stakeholders. The writing of the guiding document will be aligned with the needs of the policy-makers, law enforcements agencies and schools simultaneously.
(c) Designing and guiding document development: the planned duration of the guiding document’s development is over a period of 12 months (Table 1) [11]. The development is spread across seven activities within the implementation period, including: (1) review of literature and guidelines, (2) identifying a list of international experts, (3) developing a draft of guiding document (for the best practice on the training and utility of law enforcement in schools) and to circulate them with the experts, (4) arranging an experts meeting in Vienna (or online, pending the situation of the Novel Coronavirus 2019 pandemic travel security situation), (5) preparing a second draft of the guiding document per the feedback of the experts, (6) circulating and collecting feedback on the second draft of the guiding document using a suggested set of characteristics for the guiding document (Table 2) and (7) last round of updating the guiding document and receiving the final feedback (Table 1).

### 2.2. The Scope of the Guiding Document and Steering Committee Criteria

The scope of the guiding document will be initially focused on how to prevent drug use inside the school environment by using the law enforcement agencies (e.g., school officers). This might be expanded on their role in prevention of violence and/or crime (as proxy or mediating factors for safety inside the school environment). We established an internal steering group of UNODC (Prevention, Treatment and Rehabilitation Section; the Justice Section and the Cyber Crime unit). There will be a lead consultant responsible for consolidating and moderating the input from the different stakeholders as well as the steering committee.

The experts’ committee will be comprised of well balanced, multidisciplinary members. They will include: (i) relevant technical experts, (ii) representatives of groups most affected by the guideline (e.g., police, schools, parents and students’ representatives), and (iii) methodologists (i.e., experts in assessing evidence and developing guidelines). We will use a mixed method approach to identify and select the key stakeholders, including the Delphi method [13], literature review and known experts in the field of crime prevention within schools.

### 2.3. Guiding Document Development and Committee Members Selection Process

During the first meeting, the technical working group will be asked to identify a main author for the guiding document, ideally it will be someone with a long-term experience in guiding document development. The content knowledge of police training, implication and programs related to crime/substance use prevention is a core asset. The content experts might be with strong views. Aside from the main author, a chair of the technical working group will be selected based on the strength in facilitating group discussions and interpreting evidence. All disagreements will be diverted to the aforementioned internal steering committee to arbitrate and align with the existing UN standards and guidelines. [11].

When it comes to the technical experts, they will be selected based on their expertise either in the field of substance and/or crime prevention in schools and law enforcement agency training. The group will be balanced in terms of their variety of expertise and affiliations. The development of the guidelines will be within a relatively short period of time (Table 1). Therefore, we will ensure that all committee members understand their roles and the expected outcomes by providing clear information about how the meetings will be run (this includes scope, roles, tasks and processes).

### 2.4. Declaration of Conflict of Interests

A conflict of interest can occur when a set of conditions in which professional judgment concerning a primary interest (e.g., the validity of the research) might be influenced by a secondary interest (e.g., financial gain). However, the declaration of a secondary interest does not necessarily imply the presence of a conflict of interest that prevents participation in the guideline development committee. Furthermore, some members with certain expertise, might share their own experience(s) based on their own programs (either developed or applied). Therefore, the steering committee (being a UN entity working on standards and guidelines) will play an important role in mediating this input to reflect the input most relevant and constructively building the guidelines.

Research manuscripts reporting large datasets that are deposited in a publicly available database should specify where the data have been deposited and provide the relevant accession numbers. If the accession numbers have not yet been obtained at the time of submission, please state that they will be provided during review. They must be provided prior to publication.

Intervention studies involving animals or humans, and other studies that require ethical approval, must list the authority that provided approval and the corresponding ethical approval code.

## 3. Results

This initial scoping review included 17 peer-reviewed journal articles that investigated the impact of law enforcement officers (L.E.O.) in the school environments and on the students in terms of substance use and/or other criminal activities. The identified articles were categorized into four groups based on their results: (i) negative or null effect, (ii) positive effect, (iii) mixed effect and (iv) those that were unable to make definite conclusions (Table 3).

### 3.1. Negative Effect

A total of 6/11 studies reported negative effect include the following: Gottfredson et al., 2020, Ryan et al., 2018, Fisher et al., 2016, Schlosser et al., 2014, Na et al., 2013 and Sloboda et al., 2009 [14,16,17,18,19,20]. One study, Javdani, 2019 [15], reported both negative and null/no effect 4/11 studies reported null effect (Gist, 1995, Lynam et al., 1999, West and O’Neal, 2004 and Pan and Bai, 2009) [21,22,23,24]. Most of the studies were conducted in the USA. The studies that reported negative effects highlighted different types of negative impacts that police introduction to schools have on students and their attitudes. Studies that reported null effects signified that introduction of substance use prevention interventions and L.E.O. into schools have no significant effect on students and their attitude to substance use. Fisher et al., 2016 [17] reported that most of the studies included in their systematic review lack the necessary rigor for drawing strong conclusions, so the results of the study should be interpreted with care.

Lynam et al. (1999) [23] examined the impacts of project Drug Abuse Resistance Education (DARE) 10 years after administration. A total of 1002 individuals, who received either DARE or a standard drug-education curriculum in grade 6 were reevaluated at the age of 20. The results of the study showed that there were no significant differences between the two groups examined in term of actual drug use, drug attitudes and self-esteem.

West and O’Neal, 2004 [22] conducted a meta-analysis in order to measure the effectiveness of project DARE in preventing alcohol, tobacco and illicit drug use among school-aged youth. Using research findings from 11 previous studies, the authors created an overall effect size for DARE outcome evaluations reported in the included articles. The results showed that the overall weighted effect size for the included studies were very small and non-significant. Based on the results, the authors concluded that DARE intervention is ineffective.

Pan and Bai (2009) [21] seek to assess the effectiveness of DARE programme in the USA. The study made use of updated studies on DARE programme. The authors analyzed the studies characteristics that are related to the outcomes of the DARE programme on drug use and psychosocial behaviour. The results of the meta-analysis showed that the effects of DARE programme on drug use were homogenous but less than small while its effect on psychosocial behaviour were heterogeneous and not significant.

### 3.2. Positive Effect

Hammond et al. (2008) [25] conducted a meta-analysis study that was targeted at exploring the attitude of students to L.E.O. The function of L.E.O. was as instructors, including a school-based substance abuse prevention programme. The study made use of data survey from 6069 adolescent students from public schools in six metropolitan areas (New Orleans, Los Angeles, Houston, Detroit, Newark and St Louis) across USA. The survey data used for the study was drawn from Adolescent Substance Abuse Prevention Study (ASAPS), which was a randomized experimental design that made use of DARE officers as instructors. All the students included in the study (both experimental and control groups) reported that they had participated in some form of drug education programme when they were in the 9th grade. The results of the study showed that students evaluated police instructors more positively than non-police instructors.

### 3.3. Mixed Effect

A total of four studies reported mixed effect in their findings (Caputi and McLellan, 2017, Theriot et al., 2016, Bavarian et al., 2015, Theriot et al., 2009). [26,27,28,29] Caputi and McLellan (2017) [26] investigated the effectiveness and appropriateness of DARE keeping it REAL (KiR) curriculum. A total of 11 previous studies were used in this systematic review. There are concerns regarding the appropriateness of the KiR DARE programme. The authors reported the effectiveness of KiR intervention has not been established, the programme may not be appropriate for DARE’s larger audience and may not be effective in reducing substance use among elementary students. Theriot et al., 2016 [27] evaluated the impact of L.E.O. interactions on students’ attitude and their connectedness to school. They reported that this intervention did increase the positive attitude of the students towards; however, the presence of L.E.O. was also reported to reduce the students’ connectedness to school.

Bavarian et al., 2015 [28] examined whether adolescents receiving universal school-based drug-prevention programme in grade 7 varied by students’ profiles in substance use behavior post programme implementation. Data from the ASAPS were used for the study. This included four profiles of treatment students for self-reported substance use and programme recall at grade 7 (No use, Recall; No use, No Recall; Use, Recall, and Use, No Recall). Furthermore, they assessed the differences in substance use (alcohol, tobacco and marijuana) among students’ profiles from grade 7 to grade 11. The results of the study showed that students who have no baseline substance use and have programme recall are unlikely to engage in substance use. Students in the remaining three profiles are more likely to engage in substance use. Theriot et al. (2009) [29] investigated the impact of school L.E.O. on school-based arrest rate. A total of 28 schools in Southeastern part of USA participated in the study. The results of the study showed that there was a positive effect in term of arrest made for weapons and assault charges, where schools with L.E.O. had fewer arrest for weapons and assault charges. The findings of the study also showed that the number of arrests made for disorderly conducts at schools with L.E.O. is very high compared to those without police.

### 3.4. Indefinite Conclusions

Petrosino et al. (2012) [30] conducted a systematic review study in order to evaluate the effectiveness of non-educational policing strategies and programme in schools. The 11 previous studies were included in the systematic review; those studies took place in K12 schools in USA, UK and Canada. All of the included studies reported the utility of a specific school-based strategy that have involved L.E.O.

The authors were unable to make definite conclusions, mainly because most of these articles were considered as non-rigorous enough when weighed by evidence rating system used in justice and education. Furthermore, the authors submitted that the presently available evidence is not enough to make definite conclusions about whether using L.E.O. in schools has impact on crime and disorders in schools. The authors recommended that future research should make use of randomized control trials (RCT) in order to present top quality findings.

## 4. Discussion

Overall, the collaboration between law enforcement agencies and school-based substance use prevention programs continue to be frequently implemented. This is particularly valuable as drug control strategies are in most instances under the custody of security ministries. The relevance is accentuated in low- and middle-income countries where these security ministries are generally better financed than other concerned ministries in articulating these drug control strategies, leading to potentially larger engagement of law enforcement officers in responses.

However, the lack of substantial research and guiding documents for law enforcement in schools has resulted in incongruent actual and perceived roles of law-enforcement officers. It is clear that guidelines need to be produced in order to fully engage the positive potential law enforcement agencies may have especially in substance use prevention in schools. This is especially relevant as the preliminary review also revealed youth’s perceptions of law enforcement can be overall positively influenced by collaborative and engaging school-based programs.

The guiding document will be developed based on the synthesis of evidence and experts’ feedback. However, we should emphasize that these guiding documents aim at describing the effective ingredients that define an effective role of law enforcement agencies can play in such a response. They will be more descriptive rather than prescriptive in nature, they are as such not intended to be necessarily tested for efficacy but could however be tested for feasibility. Furthermore, we should note that the guiding documents are not meant to be developed as either reimbursement policies, performance measures, legal precedents, endorsement of specific packages or programs or as measures of certification or licensing [12]. The guiding document development is different from systematic reviews and evidence reports that identify and combine studies using explicit methods to reduce bias; however, they do not typically describe appropriate actions. The guiding documents use information from evidence reviews and other sources to make specific recommendations by including standards and linking the vigor of recommendation to the quality of the evidence. Through this guiding document, we seek to produce the optimal outcomes for the youth to promote their health, minimize the harm of substance use and reduce crime in the school environment. We should acknowledge that the literature review is limited in scope, as we have only included articles published in English. Furthermore, we have used two reviewers and consulted with experts for the sake of the development of this study protocol. However, this approach has been used by other stakeholders in the field [31,32] and was carefully developed to be robust and feasible for our study protocol, given the available resources prior to the meeting of the experts.

## 5. Conclusions

Law enforcement can potentially play an important role in the field of school-based prevention. However, no proper guidance is available on what role to use and how this can be made potentially more effective. A guiding document based on the existing literature and experiences globally, as well as networking and bridging of such experiences, would be key in reaching this objective. This proposal aims at availing these guidelines.

## Figures and Tables

**Table 1:** Timetable for the guideline’s development.

Month	Project Activities	Stated Objectives	Key Performance Indicators
1–3	Map literature and guidelines	Identify the existing science on effectiveness of interventions from law enforcement officers in the field of prevention of drug use in educational settings	Report on the analysis of existing literature developed
1–3	Identify list of experts	Establish a think-tank of experts to consult on guidelines to be produced	Contact list of experts developed
4–6	Develop a draft of guidelines to circulate to experts	Build the initial content of the guideline to establish discussion platform	Draft document produced
4–6	Arrange for a meeting in Vienna, Austria *	Ensure establishment of platform where key expert information and analysis is accounted	Meeting report with recommendations shared with experts
7–9	Draft 2 of the guidelines circulated	Avail guidelines strengthened by qualitative experiences shared by experts	Revised guidelines developed
10–12	Collecting the feedback of committee members and update the guidelines	Consolidate expert opinion around the guidelines developed	Feedback report consolidated and produced
10–12	Final approval of the guidelines	Develop peer reviewed guidelines on law enforcement implication in substance use prevention in educational setting	Guidelines draft circulated as CRP during CND 2021

* The meeting might become online, pending the situation of the Novel Coronavirus 2019 pandemic. CRP: Conference Room Paper; CND: United Nations Commission on Narcotic Drugs and Crime.

**Table 2:** Characteristics of the practice guidelines.

Topic	Description
1. Overview material	Structured abstract
2. Focus	The primary aim of the guidelines is on how to train and integrate the role of the law enforcement in schools
3. Goal	We expect to promote the health of the youth in schools, by preventing substance use through the utility of law enforcement in schools
4. Users of the guidelines	The intended users of the guidelines are law enforcement agencies operating in schools
5. Target population	Trainees: law enforcement agencies; Ultimate beneficiaries: youth in schools
6. Developer	The UNODC as the organizer, and committee of experts in the field (to be identified)
7. Funding source	Bureau of the United States International Narcotics and Law Enforcement Affairs
8. Evidence collection	Literature search and experts feedback
9. Grading criteria	Method for grading recommendation strength and rating evidence quality (to be identified by the experts)
10. Evidence synthesis	The utility of the evidence to create recommendations (to be identified by the experts)
11. Preliminary draft	This is an early version of the guidelines that will be circulated and reviewed by the experts
12. Updated draft	This is the updated draft to be sent for final approval by the experts
13. Definitions	List of definitions of critical terms
14. Recommendations and rationale	The list of recommended actions and to link them to support the evidence
15. Benefits and harms	Potential benefits and risks associated with the list of recommendations
16. Algorithm(s)	Graphical description of the stages and decisions in the integration and training process of the law enforcement agency in schools
17. Implementation	Any anticipated barriers for the implementation process, including supplementary materials

This table is adapted and modified from the Conference on Guideline Standardization [12].

**Table 3:** Summary of the identified articles and categorized per the studies outcomes.

Studies Data Direction (Total *n* = 17)	Reference
Negative or null effect (*n* = 11 studies)	1. Gottfredson et al., 2020 [14] 2. Javdani, 2019; Systematic review, *n* = 28 studies. [15] 3. Ryan et al., 2018 [16] 4. Fisher et al., 2016; Systematic review and meta-analysis, *n* = 7 studies [17] 5. Schlosser et al., 2014 [18] 6. Na et al., 2013 [19] 7. Sloboda, 2009 [20] 8. Pan and Bai, 2009. Systematic review, *n* = 20 studies [21] 9. West and O’Neal, 2004. Systematic review, *n* = 11 studies [22] 10. Lynam et al., 1999 [23] 11. Gist, 1995 [24]
Positive effect (*n* = 1 study)	1. Hammond et al., 2008; Meta-analysis, *n*= 6069 adolescents included [25]
Mixed effect (*n* = 4 studies)	1. Caputi and McLellan, 2017; Systematic review, *n* = 11 studies [26] 2. Theriot et al., 2016 [27] 3. Bavarian et al., 2015 [28] 4. Theriot et al., 2009 [29]
Indefinite conclusion (*n* = 1 study)	1. Petrosino et al., 2012; Systematic review, *n* = 11 studies) [30]

## Data Availability

The data presented in this study are available on request from the corresponding author. The data are not publicly available due to legal and privacy issues.
